# Phylogenetic tests reject Emery's rule in the evolution of social parasitism in yellowjackets and hornets (Hymenoptera: Vespidae, Vespinae)

**DOI:** 10.1098/rsos.150159

**Published:** 2015-09-02

**Authors:** Federico Lopez-Osorio, Adrien Perrard, Kurt M. Pickett, James M. Carpenter, Ingi Agnarsson

**Affiliations:** 1 Department of Biology, University of Vermont, Room 120A Marsh Life Science Building, 109 Carrigan Drive, Burlington, VT 05405, USA; 2 Division of Invertebrate Zoology, American Museum of Natural History, Central Park West at 79th Street, New York, NY 10023, USA; 3 Department of Entomology, National Museum of Natural History, Smithsonian Institution, Washington, DC 20004, USA

**Keywords:** social parasitism, Emery's rule, social insects, phylogeny, Vespinae

## Abstract

Social parasites exploit the brood-care behaviour and social structure of one or more host species. Within the social Hymenoptera there are different types of social parasitism. In its extreme form, species of obligate social parasites, or inquilines, do not have the worker caste and depend entirely on the workers of a host species to raise their reproductive offspring. The strict form of Emery's rule states that social parasites share immediate common ancestry with their hosts. Moreover, this rule has been linked with a sympatric origin of inquilines from their hosts. Here, we conduct phylogenetic analyses of yellowjackets and hornets based on 12 gene fragments and evaluate competing evolutionary scenarios to test Emery's rule. We find that inquilines, as well as facultative social parasites, are not the closest relatives of their hosts. Therefore, Emery's rule in its strict sense is rejected, suggesting that social parasites have not evolved sympatrically from their hosts in yellowjackets and hornets. However, the relaxed version of the rule is supported, as inquilines and their hosts belong to the same *Dolichovespula* clade. Furthermore, inquilinism has evolved only once in *Dolichovespula*.

## 1. Introduction

Division of labour and elaborate brood care are hallmarks of insect societies [1,2]. Societies of ants, bees and wasps typically comprise a reproductive queen, sterile (or less reproductive) workers and males. The worker caste specializes in provisioning the larvae and foraging, among other tasks [3]. Cooperative brood care not only underlies the success of social hymenopterans, but is also vulnerable to exploitation. For example, lycaenid butterfly larvae employ chemical and sound mimicry to dupe worker ants into carrying them into the brood chambers of the ant nests, where the workers feed the caterpillars [4–6]. This type of exploitation may be more easily enabled between close relatives because of their compatible communication systems and kin recognition cues. In an intriguing offshoot of sociality, socially parasitic hymenopterans have evolved a variety of strategies to deceive other species into caring for their young [7–19]. Queens of facultative social parasites generally usurp established nests, kill the resident queen and produce workers to gradually replace the host worker force. By contrast, most obligate social parasites, or inquilines, lack the worker caste altogether. Inquiline queens, unable to found their own colonies, invade the nests of other species and trick the conquered occupants into raising the parasitic brood, which develops into queens and males.

The evolution of social parasitism has been linked with close phylogenetic relationships. Motivated by the observed morphological affinities between parasitic species and their hosts, Emery [20] conjectured that socially parasitic ants are more closely related to their hosts than to any other species. This generalization, which has since become known as Emery's rule, has been explained according to two evolutionary scenarios. On the one hand, the intraspecific or sympatric speciation hypothesis proposes that social parasites may originate directly from their hosts [10,14,21]. Alternatively, the interspecific or social deception hypothesis claims that two species may evolve from geographically isolated populations (i.e. allopatrically) and parasitic habits develop when the populations come back together [1,12,22]. In testing these two hypotheses, finding that social parasites and their hosts are sister taxa would be a necessary condition for invoking sympatric speciation, and lack of immediate common ancestry between social parasites and their hosts would be sufficient to rule out sympatric speciation. The validity of the sympatric speciation model of social parasitism remains contentious, with studies of certain ants favouring the model [23–25], and absence of support for Emery's rule in other social Hymenoptera [22,26–35]. Some of the latter studies, however, support a relaxed version of Emery's rule, that is, parasites and hosts are close relatives, but not sister taxa.

Phylogenetic analyses of inquiline wasps and their hosts seldom support the strict form of Emery's rule, instead finding that inquilines are monophyletic [29–32]. In social wasps, parasitic behaviour has been documented in paper wasps (Polistinae), and yellowjackets and hornets (Vespinae). The subfamily Vespinae, among its 70 recognized species, includes five species of inquilines and two facultative social parasites, most of which occur in the yellowjacket genera *Dolichovespula* and *Vespula*. Two previous studies have assessed the veracity of Emery's rule in yellowjackets. First, Varvio-Aho *et al*. ([36], see also [37]) analysed allozymes from eight species and reported that the inquilines *Vespula austriaca* and *Dolichovespula omissa* were sister to their hosts, therefore, supporting Emery's rule. Upon reanalysis of Varvio-Aho *et al*.'s [36] data, however, Carpenter [38] found that the characters were largely uninformative and *D. omissa* was not sister to its host. Second, Carpenter & Perera [32] performed a cladistic analysis of yellowjackets based on morphological and behavioural characters and recovered the inquilines *Dolichovespula adulterina* and *D. omissa* as sister taxa, thus rejecting Emery's rule. Similarly, the obligate and facultative social parasites of *Vespula* were not sister to their respective hosts [32].

However, these previous phylogenetic studies of parasites and their hosts in vespine wasps were based on relatively few data and lacked resolution. For example, the analysis of Carpenter & Perera [32] resulted in an inquiline clade as part of a large polytomy with other *Dolichovespula* species. A robust and well-resolved phylogeny is essential for elucidating the evolution of predisposing traits that may explain why inquilinism occurs primarily in certain taxa. Such traits can be size of reproductives, nest-mate recognition signals [39], mating frequency [40] and sterility-inducing queen pheromones [41], to name a few. Here, to our knowledge, we carry out the first molecular phylogenetic analysis of social parasites and their hosts in yellowjackets and hornets. Our study includes the inquilines *D. adulterina*, *Dolichovespula arctica* and *D. omissa*, and the facultative social parasites *Vespula squamosa* and *Vespa dybowskii*. These are five of the seven known social parasites in the Vespinae. We infer the relationships among these taxa and their hosts based on the analysis of 12 gene fragments to test two mutually exclusive hypotheses. First, social parasites evolved sympatrically from their hosts, and therefore, Emery's rule in its strict sense is applicable in vespine wasps. Second, inquilinism has evolved only once in *Dolichovespula*, and thus the three inquiline species of *Dolichovespula* are monophyletic. Moreover, we discuss our results in terms of a ‘relaxed Emery's rule’ in which for any clade of social parasites the most closely related outgroup clade includes the host species [10,27].

## 2. Material and methods

### 2.1 Taxonomic sampling

We assembled a set of 38 species from all genera in the Vespinae and spanning the distribution range of the subfamily. We included the following parasitic species and their hosts, which are enclosed in parentheses: the Palearctic *D. adulterina* (*Dolichovespula saxonica*, *Dolichovespula norwegica*; [42,43]), *D. omissa* (*Dolichovespula sylvestris*; [42]), and *V. dybowskii* (*Vespa simillima*, *Vespa crabro*; [44,45]) and the Nearctic *D. arctica* (*Dolichovespula arenaria*, *Dolichovespula alpicola*; [46–50]) and *V. squamosa* (*Vespula maculifrons*, *Vespula vidua, Vespula flavopilosa*, *Vespula germanica*; [51–55]).

### 2.2 DNA extraction, amplification and sequencing

Extraction, amplification and sequencing protocols follow Lopez-Osorio *et al*. [56]. Briefly, we extracted genomic DNA using the DNeasy Blood & Tissue Kit (Qiagen) and conducted PCR amplification using PuReTaq Ready-To-Go PCR Beads (GE Healthcare). We sequenced fragments of seven mitochondrial genes and five nuclear markers: 12S and 16S ribosomal DNA (12*S*, 16*S*), cytochrome oxidase I and II (*COI*, *COII*), ATPase subunits 8 and 6 (*ATP8*, *ATP6*), cytochrome *b* (*Cytb*), 28S ribosomal DNA D2-D3 expansion regions (28*S*), elongation factor 1 alpha F2 copy (*EF1*), RNA polymerase II (*Pol II*), wingless (*Wg*) and rudimentary (*CAD*). Three of these genes (*CAD*, *ATP8* and *ATP6*) were not used in Lopez-Osorio *et al*. [56]. We amplified *CAD* with primers CD892F and CD1491R from Ward *et al*. [57] and developed primers C2-J3661 (5′-TTG GWC AAT GYT CWG AAA TTT GTG G) and A6-N4543 (5′-CCA GCA WTT ATW TTA GCT GAT AAT CG) to amplify a region spanning the mitochondrial genes *ATP8* and *ATP6*—primers were labelled according to their positions in the *Drosophila yakuba* mitogenome [58]. The PCR program for this primer pair was 35 cycles of 30 s at 94°C, 30 s at 55°C and 45 s at 72°C, preceded by 4 min at 94°C and followed by 6 min at 72°C.

Forward and reverse sequences were assembled into contigs and trimmed of low-quality ends in Geneious 6.1.7 (Biomatters Ltd). The sequences generated with the new primer pair were annotated using the MITOS WebServer [59]. Although the region amplified with primers C2-J3661 and A6-N4543 also spans the *trnK* and *trnD* genes, these sequences were not included in downstream analyses because of their short length and lack of variability. We aligned sequences with MAFFT v. 7.017 [60] using the automatic strategy selection, removed introns of *CAD* and indel regions of *ATP8* and *Wg*, and concatenated gene matrices using SequenceMatrix [61]. The concatenated alignment used in all analyses contains 418 sequences; 238 of these were previously published [56] and the remaining sequences were generated for this study (GenBank accessions KT225582–KT225591, KT250513–KT250524, KT257109–KT257164 and KT273417–KT273481).

### 2.3 Phylogenetic analyses

We performed parsimony analyses of single genes and the concatenated data using TNT [62]. The search strategy in all cases consisted of 5000 replicates using random sectorial searches, drifting, ratchet and fusing combined (xmult=rss fuse 5 drift 5 ratchet 10). In all searches, gaps were treated as missing data. Group support was calculated with 5000 replicates of symmetric resampling and the results were summarized with group present/contradicted (GC) frequencies.

We employed three partitioning strategies in maximum-likelihood and Bayesian analyses of the concatenated data: (i) assigning each gene to a separate subset; (ii) defining each codon position in each protein-coding gene as a character set, in addition to three blocks of rDNA genes, resulting in 30 subsets (see the electronic supplementary material, table S1); and (iii) submitting these 30 predefined subsets to PartitionFinder v. 1.0.1 [63] to find the best-fit partitioning scheme and choose substitution models. In the greedy search with PartitionFinder, branch lengths were set to unlinked, 56 different models were compared for each subset and models were selected according to the Akaike information criterion corrected for sample size (AICc). In the former two partitioning methods, substitution models were chosen with the AICc as implemented in jModeltest v. 2.1.7 [64]. In all cases, when the model chosen was not compatible with MrBayes, the closest available model was used.

Maximum likelihood (ML) analyses of the concatenated data were carried out using the OpenMP and MPI versions of GARLI v. 2.01 [65]. ML analyses consisted of 100 search replicates with default settings except for topoweight=0.01 and brlenweight=0.002. These two deviations from default settings were also employed in ML bootstrap analyses, which consisted of 500 pseudoreplicates.

Bayesian analyses of single genes and the concatenated data were conducted using MrBayes v. 3.2.3 [66] on CIPRES [67] with nucmodel=4by4, nruns=2, nchains=8 and samplefreq=1000. Unconstrained Markov chain Monte Carlo analyses were run for 40 M generations using the different partitioning schemes, whereas constrained analyses (see below) were carried out for 20 M generations employing the character subsets identified by PartitionFinder. Base frequencies, substitution rates, the gamma shape parameter and proportion of invariable sites were unlinked across subsets. We set a shorter prior on the mean branch length—brlenspr=unconstrained:exp(100)—to address the long-tree problem of partitioned analyses in MrBayes [68]. We assessed convergence by examining effective sample size (ESS) values with Tracer v. 1.6 [69] and the potential scale reduction factor for all parameters in MrBayes. In all analyses of the concatenated data, stationarity was reached in less than 4 million generations.

### 2.4 Constraint analyses and topology tests

We conducted constraint analyses to quantify the difference in likelihoods between unconstrained and constrained topologies. Eight constraints enforcing host–parasite monophyly were evaluated: each social parasite sister to its primary host in separate topologies, resulting in five constraint trees; all five parasites sister to their respective hosts; all inquilines sister to their corresponding hosts and an unresolved clade of inquilines and hosts. Mean marginal likelihoods of unconstrained and constrained models were calculated using stepping-stone sampling [70] in MrBayes and employing the partitioning scheme identified by PartitionFinder. Stepping-stone analyses consisted of 31 M total cycles, four independent runs of four parallel chains each, sampling every 1000 generations and using 30 steps to yield 1000 samples within each step (*α*=0.4). The first 25% samples of each step were discarded as burn-in. Log-likelihoods were compared using Bayes factors [71] calculated as 2(*H*_0_−*H*_*A*_), where *H*_0_ and *H*_*A*_ are the log-likelihoods of the unconstrained and constrained outcomes, respectively.

## 3. Results

### 3.1 Phylogenetic relationships

**Figure 1.**
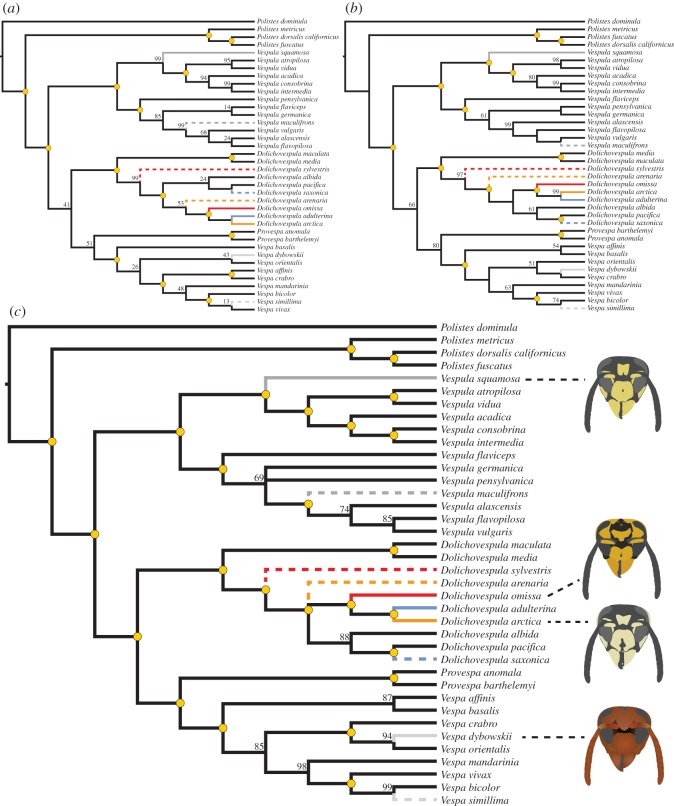
Phylogenetic relationships of social parasites, their hosts and other vespines based on the concatenated data: (*a*) single most parsimonious tree and GC values; (*b*) maximum-likelihood tree and bootstrap frequencies; (*c*) Bayesian consensus tree and clade posterior probabilities. ML and Bayesian results obtained using the best-fit partitioning scheme. Yellow dots indicate node support equal to 100. Coloured and grey solid branches indicate inquiline species and facultative social parasites, respectively. Dashed branches matching in colour indicate the corresponding hosts.

The entire DNA sequence alignment included 6568 sites and 30% of these were parsimony-informative (table 1). The best-fit partitioning scheme identified by PartitionFinder consisted of eight subsets (table 2). We found that phylogenetic relationships were stable across methods of phylogenetic inference and partitioning strategies, although with varying levels of group support (figure 1). Regardless of method of analysis or partitioning scheme, the strict form of Emery's rule was rejected in yellowjackets and hornets (figure 1). Likewise, a looser form of Emery's rule in which for any clade of parasites the nearest nonparasitic outgroup is a clade of host species [10,27] was not supported. Instead, the hosts of inquilines were scattered within a clade sister to *Dolichovespula maculata* and *D. media* (figure 1). Inquilines were consistently recovered as monophyletic with strong support—Bayesian posterior probability (PP), ML bootstrap frequency (BS) and GC=100 (figure 1). Moreover, the facultative social parasites *V. squamosa* and *V. dybowskii* did not share immediate common ancestry with their respective host species.

**Table 1.** Sequence characteristics of the complete data matrix and chosen substitution models. (PI, parsimony-informative.)

gene	no. sites	PI sites	model
12*S*	384	157	HKY+I+G
16*S*	532	156	GTR+I+G
28*S*	750	67	GTR+I
*CAD*	517	125	TIM1+G
*COII*	582	255	TVM+I+G
*COI*	1096	419	GTR+I+G
*Cytb*	433	197	GTR+I+G
*EF1aF2*	517	109	TrN+G
*Pol II*	814	110	TrN+I+G
*ATP6*	441	206	TVM+I+G
*ATP8*	111	80	HKY+G
*Wg*	391	91	K80+G
total	6568	1972

**Table 2.** Best-fit partitioning scheme identified by PartitionFinder.

subset	best model	subset partitions
1	GTR+I+G	12S, 16S
2	GTR+I+G	28S, *CAD* pos1, *EF1aF2* pos1, *Pol2* pos1, *wg* pos1, *wg* pos2
3	TrN+G	*CAD* pos3, *EF1aF2* pos3, *Pol2* pos3, *wg* pos3
4	TrN+I	*CAD* pos2, *COI* pos2, *EF1aF2* pos2, *Pol2* pos2
5	GTR+I+G	*COII* pos1, *COI* pos1, *Cytb* pos1
6	TVM+I+G	*COII* pos2, *Cytb* pos2, *ATP6* pos2
7	TrN+I+G	*COII* pos3, *COI* pos3, *Cytb* pos3, *ATP6* pos3, *ATP8* pos3
8	TIM+I+G	*ATP6* pos1, *ATP8* pos1, *ATP8* pos2

In the single most parsimonious tree found with the concatenated data, *D. arenaria* is sister to the inquiline clade (*D. omissa*, (*D. adulterina*, *D. arctica*)), but this group was poorly supported (GC=53; figure 1*a*). Using the best-fit partitioning scheme, the ML analysis of all data recovered the inquiline clade as sister to a group of three *Dolichovespula* species (figure 1*b*), whereas in the Bayesian consensus tree the inquilines were part of a polytomy (figure 1*c*), which included *D. arenaria* and (*D. albida*, (*D. pacifica*, *D. saxonica*)). However, *D. arenaria* was also sister to the inquiline clade in the Bayesian consensus trees using gene and codon partitions, although this grouping had low support (PP=87 and 73) (electronic supplementary material figure S1). In the case of *V. squamosa*, this facultative social parasite was sister to a clade of five species including two of its hosts, *V. vidua* and *V. flavopilosa*, but its primary host, *V. maculifrons*, was grouped with another species group (figure 1). Similarly, the facultative parasite *V. dybowskii* was placed in a clade separate from its main host, *V. simillima*; although *V. dybowskii* was sister to another host species, *V. crabro*, in the ML result.

### 3.2 Hypothesis testing

Interpretation of Bayes factors follows Kass & Raftery [71], and thus values greater than 150 indicate very strong evidence against the constrained topologies (table 3). The comparisons of the observed topology with those forcing host–parasite monophyly indicated that the evidence was strongly against all the alternative hypotheses.

**Table 3.** Stepping-stone estimates of marginal likelihoods and Bayes factors estimated as 2(*H*_0_−*H*_*A*_), where *H*_0_ and *H*_*A*_ are the log-likelihoods of the unconstrained topology (−44 246.01) and an alternative hypothesis, respectively.

	constraints (*H*_*A*_)	lnL	Bayes factors
	(*D. adulterina*, *D. saxonica*)	−44 688.47	884.92
	(*D. omissa*, *D. sylvestris*)	−44 332.11	172.2
	(*D. arctica*, *D. arenaria*)	−44 364.99	237.96
	(*V. dybowskii*, *V. simillima*)	−44 366.16	240.3
	(*V. squamosa*, *V. maculifrons*)	−44 540.12	588.22
	(*D. adulterina*, *D. saxonica*), (*D. omissa*, *D. sylvestris*), (*D. arctica*, *D. arenaria*), (*V. dybowskii*, *V. simillima*), (*V. squamosa*, *V. maculifrons*)	−45 202.14	1912.26
	(*D. adulterina*, *D. saxonica*), (*D. omissa*, *D. sylvestris*), (*D. arctica*, *D. arenaria*)	−44 789.44	1086.86
	(*D. adulterina*, *D. saxonica*, *D. omissa*, *D. sylvestris*, *D. arctica*, *D. arenaria*)	−44 538.58	585.14

## 4. Discussion

This study shows that social parasites among yellowjackets and hornets are not the closest relatives of their hosts, therefore, rejecting Emery's rule in its strict form. Furthermore, monophyly of *Dolichovespula* inquilines, suggesting a single origin of the parasitic strategy in this genus, is strongly supported by all our analyses. In contrast to the results of Carpenter & Perera [32], we find that the inquiline clade is not sister to *D. sylvestris*. Instead, *Dolichovespula* inquilines may be more closely related to either *D. arenaria* or a clade encompassing *D. albida*, *D. pacifica* and *D. saxonica* (figure 1). Inquiline monophyly has also been found in *Polistes* paper wasps [29,31]. Vespine parasites usually usurp host societies by means of physical combat and kill the resident queen, whereas paper wasps employ chemical camouflage and coexist with the host queen [16,72,73], but these alternative usurpation strategies have resulted in the same pattern of inquiline monophyly. Our study adds to a growing body of examples where intraspecific or sympatric speciation has not occurred in the evolution of social parasitism (e.g. [27–29,31–35]). In no case parasite and host formed a monophyletic group (figure 1). Thus, our analyses suggest that speciation occurred independently of the evolution of social parasitism. Berlocher [74] argues that observing all possible intermediate forms of parasitism may be used to test hypotheses of allopatric speciation. These intermediate forms may be intra- and interspecific usurpation [46]. In vespines, queens usurp nests of the same species as well as different species [75,76], but the latter type of usurpation is much less frequent. Within *Dolichovespula*, *D. arenaria* usurps *Vespula vulgaris* [77]. Thus, it is possible that inquilinism in *Dolichovespula* evolved from facultative, temporary usurpation in *D. arenaria* (figure 1).

In addition to lack of phylogenetic support, the characteristics of yellowjacket societies seem incompatible with a key condition of the sympatric route to new inquiline species, namely the presence of multiple laying queens per colony (i.e. polygyny) [7,14,23,78]. Certain authors (e.g. [7,8,79]) argue that polygyny might be a precursor of social parasitism because it would provide the opportunity for some queens to specialize in producing reproductives, while other queens focus on producing workers. Furthermore, the adoption of conspecific young queens resembles the series of events in nest usurpation by socially parasitic queens. Yellowjacket colonies, however, typically include a single queen and have annual cycles [75,80], and polygyny is a rare deviation restricted to large-colony species of *Vespula* in warm climates; for example, *V. germanica*, *Vespula pensylvanica*, *V. vulgaris*, *V. maculifrons* ([76] and references therein). But the phylogenetic distribution of social parasitism shows that inquilinism is mostly limited to species of *Dolichovespula* (figure 1). If polygyny enables the sympatric speciation route in the evolution of social parasitism, more social parasites that follow Emery's rule would be expected in *Vespula*.

However, the tolerance of multiple egg-laying queens in large-colony species of *Vespula* may be associated with an increased vulnerability to parasitism by *V. squamosa*, which usurps several large-colony species. *Vespula squamosa* is considered a species crossing the threshold from free-living to parasitism [52], capable of exploiting multiple host species in the *V. vulgaris* species group rather than in the more closely related *rufa* group (figure 1, see also [32,56]). This suggests that strong phylogenetic affinities may not be imperative to pass easily through the defences of host species by *V. squamosa*. It may be possible that social parasitism begins as a generalist strategy followed by host specialization. However, any events occurring after the origin of parasitism can confound inferences based on phylogenetic relationships and present associations of extant hosts and parasites [81]. A factor that has been thought to explain the rampant parasitism exerted by *V. squamosa* is its delayed release from diapause and subsequent spreading into the ranges of potential hosts [46].

Although Emery's rule in its strict form is rejected for vespines, relatively close phylogenetic relationships seem to play a key role in the evolution of social parasitism, particularly for inquilines and their hosts nested within the same *Dolichovespula* clade (figure 1). Social parasitism in the Hymenoptera involves the exploitation not only of brood care but also the colony's intricate social structure. A mixed society thus must have compatible communication systems and pheromones for nest-mate recognition [7] as well as similar mechanisms of queen control. Cell-construction may be a trait of particular importance in the evolution of inquilinism in yellowjackets. In vespines, caste differentiation is physiologically determined, and eggs destined to become queens typically develop in large cells. Cell size may function as a cue for workers to provide more food to certain larvae, which are thus launched on a queen developmental pathway [82]. For example, in honeybees, larvae housed in royal cells are maintained on a diet of royal jelly, and its major active factor, royalactin, induces their development as queens [83]. If the colony's queen in part controls the construction of large cells, the parasitic queen must be able to mimic or circumvent this aspect of the host queen's behaviour to avoid the production of workers [76].

With the exception of *D. arctica* [47], social parasites in Vespinae rely on physical attacks to subdue the host queen and her colony, but the mechanisms preventing the removal of parasitic eggs are largely unknown. Acceptance of parasitic eggs may be achieved by means of chemical mimicry, such as in the ant *Polyergus breviceps* [84]. Alternatively, parasitic eggs may be tolerated owing to lack of cuticular chemicals or use of deterrents [85–87]. To our knowledge, only a single study has investigated the chemical characteristics of parasitic eggs in vespines. Martin *et al*. [88] identified compounds from the surface of eggs of *V. dybowskii* and suggested that this species employs a chemical transparency strategy. That is, parasitic eggs of *V. dybowskii* contain external chemicals that are either undetected or unused as recognition cues. Furthermore, these authors found that the chemical profile of *V. dybowskii*, including adults, shows more significant differences in comparison to its main host, *V. simillima*, than to *V. crabro* [88]. Therefore, chemical mimicry does not seem to be involved in the parasitism of *V. simillima* by *V. dybowskii*. The similarities in chemical profiles in Martin *et al*. [88] reflect the relationships recovered in our Bayesian analysis (figure 1*c*), in which *V. crabro*is sister to *V. dybowskii* plus *V. orientalis*, but *V. simillima* is in a separate clade (see also [89]).

Our results indicate that the strict form of Emery's rule does not hold for yellowjackets and hornets, but it is clear that close phylogenetic relationships, especially in inquilines, are important in the evolution of social parasitism (figure 1). Moreover, the monophyly of inquilines of *Dolichovespula* suggests an underlying genetic basis of socially parasitic habits. Although the sympatric speciation model has been supported in certain groups of ants, such as *Myrmica* and *Mycocepurus* [23,24], it seems implausible for yellowjackets and hornets, as is the case for other social hymenopterans [26–35]. Emery's rule is commonly interpreted as a broad generalization about the evolution of a trait regardless of specific preconditions. The use of Emery's rule as something that applies under all circumstances, however, should be reconsidered. Perhaps giving more attention to the background social structure of the species for which Emery's rule holds well can help narrow down the rule's applicability.

## Supplementary Material

Figure S1. Result of Bayesian analysis using codon partitioning Table S1. Data partitioning by gene and codon position

## Data accessibility

The concatenated alignment and phylogenetic trees are available from Dryad (http://dx.doi.org/10.5061/dryad.jd038).
