# Supplementary material for: Phylogenetic tests reject Emery's rule in the evolution of social parasitism in yellowjackets and hornets (Hymenoptera: Vespidae, Vespinae)
Source: R Soc Open Sci. 2015 Sep 2;2(9):150159. doi: 10.1098/rsos.150159 (PMC4593675; doi:10.1098/rsos.150159)
Supplement: Figure S1. Result of Bayesian analysis using codon partitioning Table S1. Data partitioning by gene and codon position [file rsos150159supp1.docx]

Electronic supplementary material

Table S1. Thirty character subsets defined by gene and codon position and their corresponding substitution models.

|  | Subset | AICc Model |
| --- | --- | --- |
| 1 | 12S | HKY+I+G |
| 2 | 16S | GTR+I+G |
| 3 | 28S | GTR+I |
| 4 | CAD pos3 | HKY+G |
| 5 | CAD pos1 | K80+I |
| 6 | CAD pos2 | HKY |
| 7 | COII pos1 | TrN+G |
| 8 | COII pos2 | TrN+G |
| 9 | COII pos3 | HKY+G |
| 10 | COI pos3 | TrN+I+G |
| 11 | COI pos1 | GTR+I+G |
| 12 | COI pos2 | TVM+I |
| 13 | Cytb pos3 | HKY+G |
| 14 | Cytb pos1 | HKY+G |
| 15 | Cytb pos2 | HKY+G |
| 16 | EF1aF2 pos3 | HKY+G |
| 17 | EF1aF2 pos1 | F81 |
| 18 | EF1aF2 pos2 | JC |
| 19 | Pol II pos2 | F81 |
| 20 | Pol II pos3 | HKY+G |
| 21 | Pol II pos1 | TrN |
| 22 | ATP6 pos1 | HKY+G |
| 23 | ATP6 pos2 | HKY+G |
| 24 | ATP6 pos3 | HKY+G |
| 25 | ATP8 pos1 | TIM1+I+G |
| 26 | ATP8 pos2 | TrN+G |
| 27 | ATP8 pos3 | TrN+G |
| 28 | Wg pos3 | K80+G |
| 29 | Wg pos1 | JC |
| 30 | Wg pos2 | JC |
